# Supplementary material for: Content-rich biological network constructed by mining PubMed abstracts
Source: BMC Bioinformatics. 2004 Oct 8;5:147. doi: 10.1186/1471-2105-5-147 (PMC528731; doi:10.1186/1471-2105-5-147)
Supplement: Additional File 2 — The original results of the above study (non-essential files are deleted to keep the file size under the limit set by BMC bioinformatics). [file 1471-2105-5-147-S2.bz2 › chilibotAdditionalFile2/dip30/456ID10733106E10473/html/CCR3_CCL13.html]

 


 **CCR3** and **CCL13** 
  
Found 39 abstracts in PubMed, retrieved 30.  
 

 What does Google say? 
 PDF only 
| .edu only 

---

**Interactive relationship** (e.g. stimulation, inhibition, etc)

**Inhibitory relationship**- The compounds were potent inhibitors of eotaxin and MCP 4  [ **CCL13** ]  induced Ca 2 mobilization in RBL 2H3  **CCR3**  cells and eosinophils.  Ref: 10969084 J Biol Chem, 2000
- Preincubation of eosinophils with anti  **CCR3**  Ab 7B11 completely blocked eosinophil TEM induced by eotaxin, MCP 4  [ **CCL13** ] , and RANTES.  Ref: 10725746 J Immunol, 2000
- Identification of potent, selective non peptide CC chemokine receptor 3  [ **CCR3** ]  antagonist that inhibits eotaxin, eotaxin 2, and monocyte chemotactic protein 4  [ **CCL13** ]  induced eosinophil migration.  Ref: 10969084 J Biol Chem, 2000
- I TAC, Mig, and IP10 compete for the binding of eotaxin to  **CCR3**  bearing cells and inhibit migration and Ca 2 changes induced in such cells by stimulation with eotaxin, eotaxin 2, MCP 2 monocyte chemottractant protein 2, MCP 3, MCP 4  [ **CCL13** ] , and RANTES regulated on activation normal T cell expressed and secreted.  Ref: 11110785 J Biol Chem, 2001
**Neutral relationship**- ... MCP 4  [ **CCL13** ]  exhibited a biphasic response consistent with sequential activation of  **CCR3**  at lower concentrations and CCR2 at 10 nM MCP 4  [ **CCL13** ]  and above.  Ref: 11120855 J Immunol, 2000
- These data suggest that 1 eotaxin has a role in the early 6 h recruitment of eosinophils, while eotaxin 2 and MCP 4  [ **CCL13** ]  appear to be involved in later 24 h infiltration of these  **CCR3**  cells.  Ref: 10491000 J Immunol, 1999
- **CCR3** , CCR5 and CCR2 expressing leukocyte populations were identified at sites of MCP 4  [ **CCL13** ]  expression.  Ref: 11400154 J Pathol, 2001
- Therefore, interactions of eotaxin s, eotaxin CCL11, RANTES CCL5, and MCP 1 CCL2, MCP 2 CCL8, MCP 3 CCL7, MCP 4  [ **CCL13** ]   **CCL13**  with  **CCR3**  are responsible for the recruitment of basophils, eosinophils and mast cells.  Ref: 12009564 Mol Immunol, 2002
- CC chemokine receptor 3  [ **CCR3** ]  is also activated by other promiscuous ligands such as RANTES and monocyte chemotactic protein 4  [ **CCL13** ] .  Ref: 11286622 J Invest Dermatol, 2001
- Several CC chemokines including eotaxin CCL 11, eotaxin 2 CCL 24, RANTES CCL 5, and monocyte chemotactic protein 3 MCP 3, CCL 7 and 4 MCP 4  [ **CCL13** ] , CCL 13  [ **CCL13** ]  are potent eosinophil chemotactic and activating peptides acting through CC chemokine receptor 3  [ **CCR3** ]   **CCR3** .  Ref: 10969084 J Biol Chem, 2000
- The beta chemokine receptor  **CCR3** , which recognizes the ligands eotaxin, eotaxin 2, monocyte chemotactic protein 3, MCP4  [ **CCL13** ] , and RANTES, plays a central role in this process, and antagonists to this receptor could have potential therapeutic use in the treatment of allergy.  Ref: 10640766 J Immunol, 2000
- Furthermore,  **CCR3**  transfected human embryonic kidney cells labeled with Fura 2AM exhibited a rapid rise in intracellular free calcium after stimulation with eotaxin, eotaxin 2, or MCP 4  [ **CCL13** ] , but not with MDC.  Ref: 10069890 J Allergy Clin Immunol, 1999

**Non-interactive relationship** (e.g. studied together, co-existance, homology, etc.)

- Nanomolar concentrations of UCB35625 were sufficient to inhibit eosinophil shape change responses to MIP 1alpha, MCP 4  [ **CCL13** ] , and eotaxin, while greater concentrations could inhibit the chemokine induced internalization of both CCR1 and  **CCR3** .  Ref: 10854442 J Biol Chem, 2000
- Stably transfected Cynomolgus  **CCR3**  bound human eotaxin CCL11 with similar kinetics Kd 240 pM and was responsive to human  **CCR3**  ligands eotaxin CCL11, eotaxin 2 CCL24, and MCP4  [ **CCL13** ]   **CCL13**  in Ca 2 mobilization and chemotaxis assays, thus provides a useful alternative species model system for the analysis of modulators of eotaxin  **CCR3**  induced signaling and activation.  Ref: 12421570 Cytokine, 2002
- The chemokine receptor  **CCR3**  and ligands monocyte chemoattractant protein MCP 3, MCP 4  [ **CCL13** ] , regulated upon activation normal T cell expressed and secreted RANTES and eotaxins I and II are of particular relevance for the recruitment and activation of eosinophils.  Ref: 11340325 Int Arch Allergy Immunol, 2001
- Eosinophil chemotactic chemokines eotaxin, eotaxin 2, RANTES, monocyte chemoattractant protein 3 MCP 3, and MCP 4  [ **CCL13** ] , and C C chemokine receptor 3  [ **CCR3** ]  expression in bronchial biopsies from atopic and nonatopic Intrinsic asthmatics.  Ref: 10570327 J Immunol, 1999
- Other human  **CCR3**  agonists, such as MCP 3, RANTES and MCP 4  [ **CCL13** ]  failed to increase dermal eosinophil peroxidase activity at doses up to 1 microg site 1 whereas the latter did produce a small effect at 3 microg site 1.  Ref: 10516663 Br J Pharmacol, 1999
- These ligands include the  **CCR3**  selective chemokines eotaxin and eotaxin 2 and the more promiscuous CC chemokines, MCP 4  [ **CCL13** ] , MCP 3, MCP 2 and RANTES.  Ref: 11306952 Int Arch Allergy Immunol< MedlineTA>Int Arch Allergy Immunol, 1999
- Compared with AC and NC, the numbers of EG2 cells and the cells expressing mRNA for eotaxin, eotaxin 2, RANTES, MCP 3, MCP 4  [ **CCL13** ] , and  **CCR3**  were significantly increased in AA and NAA p <.  Ref: 10570327 J Immunol, 1999
- However, degranulation was induced only by  **CCR3**  ligands, including MCP 3, MCP 4  [ **CCL13** ] , RANTES, and eotaxin.  Ref: 10984371 J Allergy Clin Immunol, 2000
- We examined the expression of eotaxin, eotaxin 2, RANTES, monocyte chemoattractant protein 3 MCP 3, MCP 4  [ **CCL13** ] , and  **CCR3**  in the bronchial mucosa from atopic AA and nonatopic intrinsic.  Ref: 10570327 J Immunol, 1999
- Our data suggest that eotaxin, eotaxin 2, MCP 4  [ **CCL13** ] , and RANTES induce eosinophil TEM via  **CCR3**  with varied potency and efficacy.  Ref: 10725746 J Immunol, 2000
